# Serum resistin is causally related to mortality risk in patients with type 2 diabetes: preliminary evidences from genetic data

**DOI:** 10.1038/s41598-017-00138-3

**Published:** 2017-03-03

**Authors:** Andrea Fontana, Lorena Ortega Moreno, Olga Lamacchia, Concetta De Bonis, Lucia Salvemini, Salvatore De Cosmo, Mauro Cignarelli, Massimiliano Copetti, Vincenzo Trischitta, Claudia Menzaghi

**Affiliations:** 1 Unit of Biostatistics, IRCCS Casa Sollievo della Sofferenza, San Giovanni Rotondo, Italy; 2 Research Unit of Diabetes and Endocrine Diseases, IRCCS Casa Sollievo della Sofferenza, San Giovanni Rotondo, Italy; 3 Unit of Endocrinology, Department of Medical and Surgical Sciences, University of Foggia, Foggia, Italy; 4 Division of Internal Medicine, IRCCS “Casa Sollievo della Sofferenza”, San Giovanni Rotondo, Italy; 5 Department of Experimental Medicine, Sapienza University of Rome, Rome, Italy

## Abstract

Resistin has been firmly associated with all-cause mortality. We investigated, whether, in patients with type 2 diabetes (T2D), this association is sustained by a cause-effect relationship. A genotype risk score (GRS), created by summing the number of resistin increasing alleles of two genome-wide association studies (GWAS)-derived single nucleotide polymorphisms (SNPs), serum resistin measurements and all-cause death records were obtained in 1,479 (403 events/12,454 person-years), patients with T2D from three cohorts, Gargano Heart Study-prospective design (n = 350), Gargano Mortality Study (n = 698) and Foggia Mortality Study (n = 431), from Italy. GRS was strongly associated with serum resistin in a non-linear fashion (overall p = 3.5 * 10^−7^) with effect size modest for GRS = 1 and 2 and much higher for GRS >3, with respect to GRS = 0. A significant non-linear association was observed also between GRS and all-cause mortality (overall p = 3.3 * 10^−2^), with a low effect size for GRS = 1 and 2, and nearly doubled for GRS ≥ 3, with respect to GRS = 0. Based on the above-reported associations, each genetic equivalent SD increase in log-resistin levels showed a causal hazard ratio of all-cause mortality equal to 2.17 (95%CI: 1.22–3.87), thus providing evidence for a causal role of resistin in shaping the risk of mortality in diabetic patients.

## Introduction

Resistin is a 12.5 kDa cysteine-rich pro-inflammatory^1–3^ and pro-atherogenic protein^4–12^, which in humans, is primarily secreted by macrophages^13^ and firmly associated with all-cause mortality in several clinical sets including type 2 diabetes (T2D)^14–25^. Given the above-mentioned background and the deleterious role of resistin on several mortality risk factors^26–28^, it is conceivable that its association with mortality rate is sustained by a cause-effect relationship. However, no studies have so far addressed this hypothesis.

Genetic variants, robustly affecting an exposure, which in turn is associated to a given outcome, are easy-to-use tools for assessing if causality underlies the association of interest^29–31^. In our case, if genetic variants strongly linked to circulating resistin levels prove to be also associated with all-cause mortality, a strong case is made in favor of a causal role of resistin on all-cause death.

Few genome-wide association studies (GWAS) on circulating resistin have been conducted so far^32–34^. Two of them were carried out in Asians and pointed to the *RETN* locus as a major determinant of resistin levels^33, 34^. In contrast, in GWAS carried out in individuals of European ancestry we have recently shown that two different single nucleotide polymorphisms (SNPs), namely rs3931020 and rs13144478, in *TYW3/CRYZ* and *NADST4* loci respectively, are associated with serum resistin^32^. Then, in order to investigate whether or not the association between resistin and all-cause mortality previously reported in European patients with T2D^14–17, 22^ is sustained by a cause-effect relationship, a genotype risk score (GRS) based on these two SNPs, was created and used as an instrumental variable. It is of note that study patients here analyzed are from the same geographical region of some non-diabetic individuals previously investigated in the GWAS on serum resistin, pointing to both rs3931020 and rs13144478^32^.

## Results

Clinical features of patients from Gargano Heart Study- (GHS)-prospective design, Gargano Mortality Study (GMS) and Foggia Mortality Study (FMS) as well as duration of follow-up and number of events are summarized in Table 1. The three samples were quite different in terms of most clinical variables (p < 0.05), but smoking habits.

**Table 1:** Clinical characteristics of study patients.

	**GHS** (n = 350)	**GMS** (n = 698)	**FMS** (n = 431)
Males (%)	238 (68.0)	344 (49.3)	224 (52.0)
Age at recruitment (yrs)	64.5 ± 8.2	61.3 ± 9.9	63.2 ± 11.6
Smokers (%)	58 (16.6)	102 (14.6)	71 (16.5)
Diabetes duration (yrs)	13.9 ± 9.2	10.3 ± 8.8	13.1 ± 10.1
BMI (kg/m^2^)	30.1 ± 4.8	31.0 ± 5.7	30.0 ± 6.1
HbA_1C_ (%), (mmol/mol)	8.6 ± 1.9, (70 ± 20.8)	8.7 ± 2.0, (72 ± 21.9)	9.1 ± 2.2, (76 ± 24.0)
Insulin (w/wo) oral agents (%)	191 (54.6)	271 (38.8)	157 (36.4)
Anti-hypertension therapy (%)	296 (84.6)	323 (46.3)	291 (67.5)
Anti-dyslipidemia therapy (%)	227 (64.8)	195 (27.9)	162 (37.6)
Resistin (ng/ml)	10.7 ± 6.7	10.1 ± 8.1	8.5 ± 6.2
Follow-up (yrs), (py)	5.4 ± 2.5; (1,890)	10.8 ± 3.5; (7,504)	7.1 ± 2.5; (3,060)
Events (n)	78	206	119
IR (n. events per 100 py)	4.1	2.7	3.9

Continuous variables were reported as mean ± SD whereas categorical variables as total frequency and percentages. GHS: Gargano Heart Study; GMS: Gargano Mortality Study; FMS: Foggia Mortality Study; BMI: body mass index; HbA1c: glycated haemoglobin; IR: incidence rate of all-cause death events; py: person-years.

In each cohort, as well as in the combined sample comprising 1,479 individuals (403 deaths/12,454 person years), each SD increase of log-resistin levels was significantly associated with all-cause mortality (Table 2). In each cohort as well as in the combined sample, such association was log linear. Similar associations were obtained after taking into account sex, age at recruitment, smoking habits, BMI, HbA1c, anti-hypertension and anti-dyslipidemia therapies, all being general risk factors shaping the risk of mortality rate (Table 2).

**Table 2:** Association between serum resistin and all-cause mortality in individual studies and in the combined sample.

	GHS (N = 350)		GMS (N = 698)		FMS (N = 431)		Combined (N = 1,479)		*Between-study heterogeneity*
	HR (95% CI)	p	HR (95% CI)	p	HR (95% CI)	p	HR (95% CI)	p	
Model 1	1.55 (1.27–1.90)	1.9 * 10^−5^	1.38 (1.21–1.57)	2.3 * 10^−6^	1.31 (1.11–1.56)	2.0 * 10^−3^	1.39 (1.27–1.53)	2.4 * 10^−12^	4.2 * 10^−1^
Model 2	1.64 (1.31–2.05)	1.5 * 10^−5^	1.30 (1.14–1.49)	6.9 * 10^−5^	1.17 (0.98–1.40)	8.0 * 10^−2^	1.31 (1.19–1.44)	2.5 * 10^−8^	2.1 * 10^−1^
Model 3	1.60 (1.26–2.02)	1.0 * 10^−4^	1.29 (1.11–1.49)	9.0 * 10^−4^	1.11 (0.92–1.33)	2.7 * 10^−1^	1.27 (1.14–1.41)^§^	2.1 * 10^−5^	7.7 * 10^−2^

GHS: Gargano Heart Study; GMS: Gargano Mortality Study; FMS: Foggia Mortality Study. HRs (95% CI) are given for the increase of 1 SD of log transformed values of serum resistin. Model 1: unadjusted in individual studies and adjusted for study sample (i.e. GHS, GMS and FMS) in the combined analysis. Model 2: adjusted for sex, age at recruitment, smoking habits, BMI and study sample in the combined analysis. Model 3: adjusted for sex, age at recruitment, smoking habits, BMI, HbA1c, anti-hypertension and anti-dyslipidemia therapies and study sample in the combined analysis. ^§^Robust 95%CI confidence interval (due to the presence of between-study heterogeneity, i.e. for p < 0.10).

In all three cohorts, both SNPs tended to be associated with circulating resistin levels, though reaching statistical significance only in GMS and FMS (Supplementary Table S1); in contrast, such association became stronger and statistically significant, in the combined sample after ascertaining the absence of a between-study heterogeneity (Supplementary Table S1).

To obtain a powerful instrumental variable (IV), able to address the causal nature of the relationship between serum resistin and all-cause mortality, an individual genotype risk score (GRS) was then created by simply summing the number of resistin increasing alleles, carried by each subject. In the combined sample, 231, 587, 599, 60 and 2 individuals carried 0, 1, 2, 3 and 4 risk alleles, respectively. Individuals carrying 3 and 4 risk alleles were then pooled and considered together for further analyses.

When evaluating the association with circulating resistin levels, GRS was treated as categorical variable because of smallest achieved Alkaike information criterion (AIC) with respect to GRS treated as continuous variable (fully adjusted AIC = 3697 vs. 3708, respectively). Indeed, categorical GRS was strongly associated with serum resistin (overall p value = 3.5 * 10^−7^), with effect size being modest for 1 and 2 and much higher for >3 risk alleles, as compared to GRS = 0 (Supplementary Table S2, left panel).

**Figure 1:**
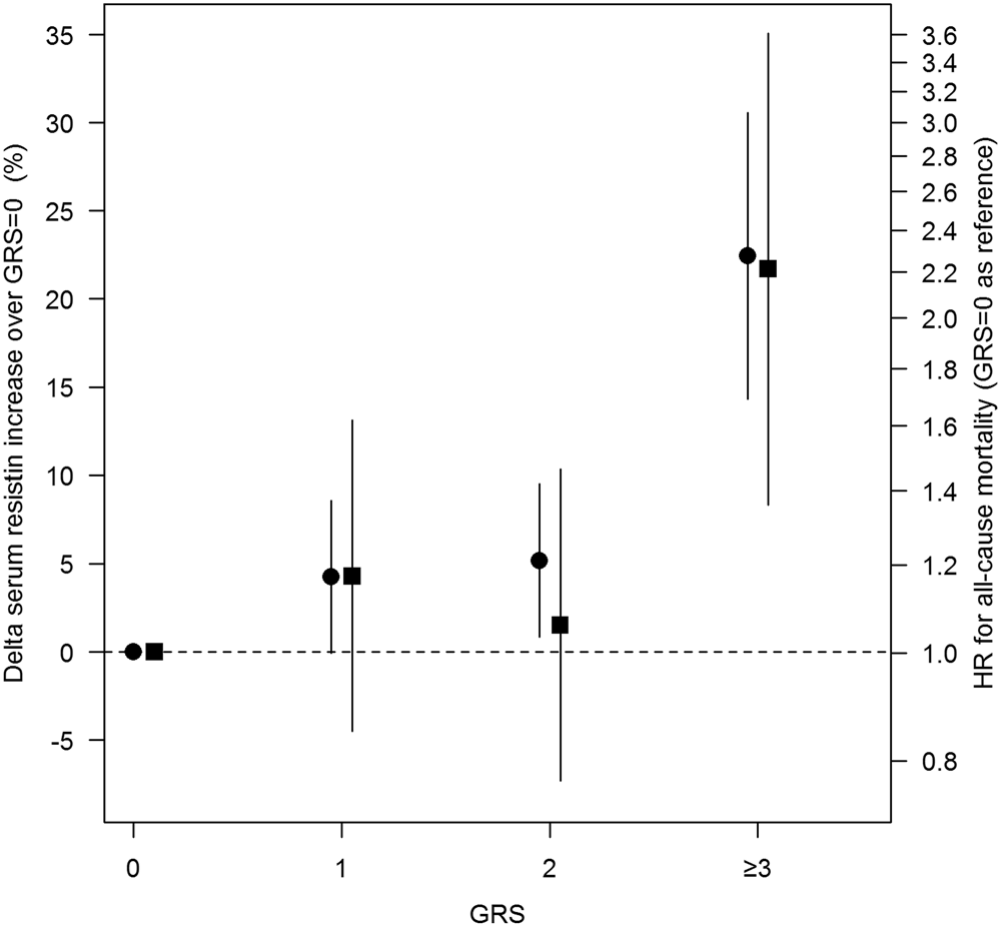
Plots of percentage changes in the estimated log-resistin means (circles) and HRs (squares), along with error bars which represented 95% CI of each percentage change at issue and of HRs, respectively. Both percentage changes and HRs were estimated taking into account GRS = 0 as the reference group. Error bars for percentage changes in log-resistin means were referred to the approximated standard errors derived using delta method (Supplementary Information).

Also when evaluating all-cause mortality, GRS was treated as categorical, rather than a continuous variable, with fully adjusted AIC being 1864 vs. 1867, respectively. Categorical GRS was associated with all-cause mortality rate (overall p value = 3.3 * 10^−2^), with effect size being modest for 1 and 2 and much higher for >3 risk alleles with respect to GRS = 0 (Supplementary Table S2, right panel). Interestingly, both serum resistin means and mortality rates increased in the same magnitude when the number of GRS risk allele increases. In fact, the effect of increasing number of risk alleles on serum resistin (expressed as percent increase vs. individuals with 0 risk alleles) paralleled that on mortality risk, (expressed as hazard ratio using individuals with 0 risk alleles as the reference group) (Fig. 1), clearly suggesting that the two associations are biologically related.

It is of note that GRS was not associated with any confounder (i.e. age at recruitment, sex, smoking habits, BMI, HbA1c, anti-hypertension and anti-dyslipidemia therapies) we accounted for, when testing the association between resistin and mortality rate (p values ranging from 0.13 to 0.64).

In subsidiary analyses, we noted that homozygotes risk allele carriers of either rs3931020 or rs13144478 had the highest effect size both on resistin levels and mortality rate as compared to the other genotype groups (data not shown), thus confirming that both SNPs contributed to the associations we here report for GRS group 3.

For each genetic equivalent standard deviation (SD) increase in log-resistin levels, a causal hazard ratio (HR) of all-cause mortality equal to 2.17 (95%CI: 1.22–3.87) was found. Such estimate was different, (fully adjusted p from Cochran Q test = 7.3 * 10^−2^), from the actual HR of the association between serum resistin and all-cause mortality (i.e. HR = 1.27; 95%CI: 1.14–1.41).

## Discussion

Several studies have repeatedly reported resistin associated with mortality rate^14–25^. In this study we have now addressed whether in patients with T2D this association is sustained by a cause-effect relationship. Our present data show that a GRS, based on genetic variants strongly associated with serum resistin in Europeans^32^, is also associated with all-cause mortality. According to the use of genetic variants as a tool to address causality^29–31^, our finding provides, to the best of our knowledge, the first evidence for a possible causal association between resistin and all-cause mortality. This scenario is further supported by the observation that the relationships of GRS with serum resistin concentration on the one hand and risk of all-cause death on the other were both non-linear and parallel to each other. The lack of linearity of such associations, as well as the one between resistin circulating levels and all-cause mortality previously reported^17^ (that, in fact, we here confirm), deserves further, specifically designed, investigations to be addressed.

Of note, the GRS we used was based on two SNPs that are not only associated with circulating resistin in Europeans, but also with resistin gene (*RETN*) mRNA levels^32^, thus reinforcing its biological meaning and in a broader sense, our study design.

As suggested by Cochran Q-test <0.10, the genetic equivalent HRs of one SD increase in log-resistin levels for all-cause mortality was higher than the observed one (i.e. 2.17 vs.1.27, an approximately threefold difference on a log scale). Such discrepancy may be explained by the difference between a totally stable genetic effect, operating since birth on the one hand and, conversely, the effect exerted by serum resistin, evaluated only for the few years of our follow-up, with presumably highly variable levels, especially in heavily treated patients as are those with T2D^35, 36^. In addition, although no genome-wide data have reported the involvement of TYW3/CRYZ and NADST4 loci in risk factors for death, (http://www.gwascentral.org/), we cannot entirely exclude that these two loci exert, beside those on serum resistin, additional, still unknown, effects, somehow related to the risk of mortality.

Although the biology underlying the association between resistin and mortality rate has not been specifically addressed in this study, one can easily speculate that it is mediated by the deleterious effect exerted by resistin on intermediate metabolism, low-grade inflammation and atherosclerotic processes^1–12^ all established mortality risk factors. Another limitation of our study is represented by the lack of C-reactive protein and/or leukocyte measurements, two established risk factors, which would have helped clarify the pathway linking resistin and mortality rate.

We like to acknowledge that a great caution is needed in interpreting our data, which though of interest and entirely novel, cannot be considered as established. In fact, although, the sample we analyzed comprises more than 1,400 individuals with a total of 403 incident cases, the p values we obtained does not allow to exclude the possibility of a false positive result.

Moreover, a clear baseline clinical heterogeneity across the three study cohorts was evident. Also mortality rate was different across samples. Despite this, no difference was observed in the resistin effect on all-cause mortality, thus making unlikely that such heterogeneity have played a role in confounding our results. Nonetheless, when running pooled analyses, we were conservative enough to adjust for “study sample”, thus taking into account all baseline differences.

In addition, very likely because of the sub-cultured, mostly rural area as the one where our cohorts have been recruited, the proportion of patients treated at time of enrollments with insulin and/or anti-hypertensive and statins were lower than hoped. We cannot exclude that these baseline conditions, which luckily enough has being slowly changing in the last few years, may have affected the results obtained.

We also acknowledge that it remains to be investigated whether our finding applies also to non-diabetic individuals and whether it is extendible to populations of non-European ancestry with different environmental and genetic background which are known to affect serum resistin concentration^37^.

In conclusion, our data strongly point to resistin as a causal risk factor for all-cause mortality in T2D. Further confirmatory studies are needed before this finding may be considered as established. Additional studies are also necessary to verify whether adding resistin to previously validated tools^38, 39^ improves the ability to predict mortality rate in T2D and to explore if treatments aimed at reducing resistin levels^36, 40, 41^ also decrease the risk of death in such patients.

## Methods

### Patients

Three cohorts of patients with T2D (ADA 2003 criteria) from Apulia, central-southern Italy have been analyzed: the GHS-prospective design^14, 15, 42–46^, the GMS^14, 15, 17, 38, 47^ and the FMS)^17, 38, 45, 47^, (see Supplementary Information for details).

Clinical data were obtained from a standardized interview and examination as previously described^14, 15, 17, 38, 42–45, 47^. Serum resistin was measured by a commercial ELISA (Bio Vendor, Brno Czech Republic) as previously described^48^ in 350 (95.2%) participants of GHS, 698 participants (67.9%) of GMS and 431 participants (37.4%) of FMS, constituting the eligible samples for the present analysis.

The study protocols and the informed consent procedures were approved by the Institutional Ethic Committee of Istituto di Ricovero e Cura a Carattere Scientifico (IRCCS) “Casa Sollievo della Sofferenza” and the University of Foggia, respectively. All participants gave written informed consent. All methods were carried out in accordance with the approved guidelines.

### Genotyping

SNPs rs3931020 and rs13144478 were genotyped by Taqman SNP allelic discrimination technique by means of an ABI 7000 (Applied Biosystems, Foster City, CA) as previously described^32, 49^. Call rate and concordance rate were ≥96 and >99%, respectively.

The SNPs were in Hardy–Weinberg equilibrium (HWE) with the exception of rs3931020, p = 0.03 in FMS.

### Statistical methods

Patients’ baseline characteristics are reported as mean ± standard deviation (SD) and percentages for continuous and categorical variables, respectively.

The relationship between resistin serum concentrations and all-cause mortality was log linear, as assessed by the Kolmogorov-type supremum test based on a sample of 10,000 simulated residual patterns^50^ and by visual inspection of residual pattern plots. Then, resistin levels were firstly normalized by a logarithm transformation and hence divided by its SD (i.e. log-resistin levels), in order to increase its clinical interpretability.

Detailed statistical methods used to assess associations between circulating resistin levels, SNPs and all-cause mortality risk, and to perform instrumental variable (IV) analysis, were reported in Supplementary Information.

Our combined sample of 1,479 patients with the observed mortality rate, achieves 80% power (assuming a type I error of 5%) to detect HRs of 1.15 and 1.19 for each unitary increase of one SD in log-resistin levels and for each unitary increase of GRS, respectively. Furthermore, this pooled sample achieves 80% power to detect a regression slope of 0.10 in SD-rescaled log-resistin levels for each unitary increase of one risk allele in GRS.

For all statistical analyses, a two-sided p-value < 0.05 was considered as significant, except when the effect of each SNP on serum resistin levels was evaluated (Supplementary Table S1). In this case, a Bonferroni-adjusted p-value < 0.025 was considered for statistical significance. All analyses were performed using SAS v.9.4 (SAS Institute, Cary, NC). Plots were produced using Comprehensive R Archive Network (CRAN) version 3.2.

## Electronic supplementary material

Supplementary information
