# Supplementary material for: Serum resistin is causally related to mortality risk in patients with type 2 diabetes: preliminary evidences from genetic data
Source: Sci Rep. 2017 Mar 3;7:61. doi: 10.1038/s41598-017-00138-3 (PMC5427821; doi:10.1038/s41598-017-00138-3)
Supplement: Supplementary file 1 — Supplementary information [file 41598_2017_138_MOESM1_ESM.pdf]

Serum resistin is causally related to mortality risk in patients with type 2 diabetes: preliminary evidences from genetic data

Andrea Fontana[1§], Lorena Ortega Moreno[2§], Olga Lamacchia[3], Concetta De Bonis[2], Lucia Salvemini[2], Salvatore De Cosmo[4], Mauro Cignarelli[3], Massimiliano Copetti[1], Vincenzo Trischitta[2,5^*], Claudia Menzaghi[2^*]

Supplementary Information

Patients

The Gargano Heart Study (GHS) and the Gargano Mortality Study (GMS) were recruited at IRCCS "Casa Sollievo della Sofferenza", San Giovanni Rotondo. The GHS consists of 368 patients with T2D and coronary artery disease consecutively recruited from 2001 to 2008 and followed yearly until 2011 for incident major cardiovascular events and all-cause mortality. The GMS comprises 1,028 patients recruited from 2000 to 2005 for a study having all-cause mortality as the end-point and followed until December 2014. The FMS comprises 1,115 patients consecutively recruited at the University of Foggia from 2002 to 2008 for a study having all-cause mortality as the end-point and followed until March 2015. Exclusion criteria were for all studies the presence of poor life expectancy for non-diabetes-related diseases.

At follow-up, the vital status of study patients was ascertained, either by telephone interview with the patient or his/her relatives or by queries to the registry office of cities of residence. In the GHS, confirmation of the event was obtained from death certificates and in the GMS, the last follow-up was carried out by queries to the Italian Health Card (http://sistemats1.sanita.finanze.it/wps/portal/portalets/cittadinots/ts).

Statistical methods

The assumption of normality distribution was checked by means of quantile-quantile (Q-Q) plots and Shapiro-Wilks test.

Linear regression models were performed to assess the effect of each single nucleotide polymorphism (SNP) (assuming an additive genetic model of inheritance) on log-resistin levels and results were reported as regression coefficients (β) along with standard errors (SE). Univariable and multivariable

linear regression models were estimated within each study and in the combined sample, respectively. Multivariable models were adjusted both for "study sample" only (i.e. sample-adjusted models) and progressively adjusted for study sample, age at recruitment, sex, smoking habits, BMI, HbA1c, anti-hypertension and anti-dyslipidemia therapies (i.e. fully-adjusted models) and were performed in an individual patient data meta-analysis fashion (1), after checking for the absence of between-study heterogeneity (i.e. the absence of a significant SNP-by-sample interaction effect) in the sample-adjusted models. Deviation from Hardy–Weinberg equilibrium (HWE) of the two SNPs was investigated by exact Wiggington χ^2 test (2).

Mortality rates were expressed as the number of new events (i.e. deaths) per total number of person-years (py). Time variable was defined as the time between the baseline examination and date of the event (i.e. all-cause mortality), or, for subjects who did not experience any event, the date of the last available clinical follow-up. To assess the association between serum resistin levels and the event occurrence, univariable and multivariable Cox proportional hazards models were estimated within each study and in the combined sample, respectively. Multivariable Cox models were adjusted both for study sample only and fully-adjusted, respectively. Pooled data analyses were performed in an individual patient data meta-analysis fashion (1), after checking for the absence of between-study heterogeneity (i.e. the absence of a significant resistin-by-sample interaction effect). Risks were reported as hazard ratios (HR) along with their 95% CI per 1-SD increase in log-resistin levels. In case of the heterogeneity was present, HR were estimated along with robust 95% CI.

Linear and non-linear relationships between genotype risk score (GRS) and log-resistin levels were evaluated by fitting a fully-adjusted linear regression (with GRS treated as a continuous covariate) and ANCOVA models (with GRS treated as categorical covariate, i.e. 0,1,2,$\geq$3 levels), respectively. The model which achieved the minimum Akaike Information Criterion (AIC) was considered to best fit the data. Furthermore, within ANCOVA models, pairwise comparisons of adjusted log-resistin means between GRS categories (GRS=0 was taken as the reference) were performed and p-values were adjusted for multiple comparisons, following Dunnett method.

Similarly, linear and non-linear relationships between GRS and mortality rate were evaluated by fitting fully-adjusted Poisson models, which included GRS as a continuous and categorical covariate and

follow-up time as the offset variable, respectively (and the model which achieved the minimum AIC was considered as it best fits data). Furthermore, pairwise comparisons of mortality rates between GRS categories (GRS=0 was taken as the reference) were performed and p-values were adjusted for multiple comparisons, following Dunnett method.

Moreover, for each GRS category, percentage changes in the estimated log-resistin means (with respect to GRS=0) were plotted besides the estimated HRs, along with error bars which represented 95% CI of each percentage change at issue and of HRs, respectively. Error bars for percentage changes in log-resistin means were estimated using the approximated standard errors derived from delta method as reported below:

Computation of approximated standard errors and 95% confidence intervals of resistin means' percentage variation between GRS categories with respect to GRS=0.

	Resistin			Percentage variation vs. GRS=0 (%)		
**GRS risk allele (N)**	**Mean**	**Variance**	**Covariance (vs. GRS=0)**	**Mean (%)**	**Approximated SE (%)***	**Approximated 95%CI***
0	3.7382	0.00681	---	0 (ref.)	0 (ref.)	0 (ref.)
1	3.8969	0.00396	0.00218	4.245 %	2.210 %	-0.086, 8.576 %
2	3.9321	0.00398	0.00221	5.187 %	2.215 %	0.845, 9.529 %
≥ 3	4.5768	0.01823	0.00181	22.433 %	4.144 %	14.312, 30.555 %

*Approximated SE and 95% confidence intervals (95%CI) were computed using the following formula:

$$\mathrm{SE}\left[\left(\frac{(\mathrm{RES}_i - \mathrm{RES}_0)}{\mathrm{RES}_0}\right)\times 100\right] \approx 100 \times \sqrt{\frac{\mu_i^2\sigma_i^2 + \mu_0^2\sigma_0^2 - 2\mu_i\mu_0\sigma_{i,0}}{\mu_0^4}} \quad \forall i = \{1,2,\geq 3\} \text{ and } |\mu_0|>0$$

where μ_i and σ_i^2 are the mean and the variance of log-resistin levels for the *i*-th non-reference GRS risk allele category (RES_i) respectively, whereas μ_0 and σ_0^2 are the mean and the variance of log-resistin levels for the reference (i.e. 0) GRS risk allele (RES_0) respectively. Finally, $\sigma_{i,0}$ is the covariance between log-resistin levels for the *i*-th and the reference GRS categories.

Such formula was algebraically derived starting from the following one:

$$\sigma_{g(T)}^2 \approx \sum_{i=1}^{k} g_i'\left(\underline{\mu}\right)^2 \sigma_{T_i}^2 + 2\sum_{i=1}^{k}\sum_{j>i}^{k} g_i'\left(\underline{\mu}\right) g_j'\left(\underline{\mu}\right)\sigma_{T_i,T_j}$$

where the delta method was applied to calculate an approximation of the variance of any differentiable function $g(.)$ of $\underline{T} = (T_1, T_2, \dots, T_k)$ random variables with means $\underline{\boldsymbol{\mu}} = (\mu_1, \mu_2, \dots, \mu_k)$

To estimate the average causal effect of serum resistin levels on all-cause mortality, an instrumental variable (IV) analysis (3, 4) was performed, using the categorized GRS as the main instrument. Specifically, having assumed that all of instrumental conditions were met (i.e. 1-the instrument must be associated with resistin levels, 2-the instrument must not affect the mortality except through its potential effect on resistin levels and 3-the instrument must be independent of the potential observed and unobserved confounding factors), the two-stage least squares (2SLS) estimator procedure was assessed: a first-stage ANCOVA model was estimated to determine, for each subject, the predictive values of log-resistin levels on the basis of GRS (treated as categorical) and study sample as covariates. Thereafter, a second-stage Cox model was fit to determine the expected (i.e. causal) HR for each SD increase in log-resistin levels, using the predicted log-resistin levels from the first-stage ANCOVA model and study sample as covariates.

A statistically significant causal HR suggests the presence of a causal relationship between serum resistin levels and all-cause mortality risk. Finally, to statistically evaluate whether the estimated causal HR was different from the estimated association HR, the Cochran Q-test was performed. In such a case, heterogeneity hold for p-values less than 0.10 (5).

References

1. **Olkin I, Sampson A** 1998 Comparison of meta-analysis versus analysis of variance of individual patient data. Biometrics 54:317-322
2. **Wigginton JE, Cutler DJ, Abecasis GR** 2005 A note on exact tests of Hardy-Weinberg equilibrium. Am J Hum Genet 76:887-893
3. **Katan MB** 1986 Apolipoprotein E isoforms, serum cholesterol, and cancer. Lancet 1:507-508
4. **Lawlor DA, Harbord RM, Sterne JA, Timpson N, Davey Smith G** 2008 Mendelian randomization: using genes as instruments for making causal inferences in epidemiology. Statistics in medicine 27:1133-1163
5. **Greenland S** 1987 Quantitative methods in the review of epidemiologic literature. Epidemiol Rev 9:1-30
